# 337. Association of atrial fibrillation burden with in-hospital mortality in patients with sepsis: Analysis of MIMIC-III database

**DOI:** 10.1093/ofid/ofad500.408

**Published:** 2023-11-27

**Authors:** Yongseop Lee, Jihoon Seo, Min Han, Sangmin Ahn, Jung Ah Lee, Jung Ho Kim, Jin Young Ahn, Su Jin Jeong, Nam Su Ku, Jun Yong Choi, Joon-sup Yeom, Se Yoon Park, Dukyong Yoon

**Affiliations:** Yonsei University School of Medicine, Seoul, Seoul-t'ukpyolsi, Republic of Korea; Dept. of Biomedical Systems Informatics, Yonsei University College of Medicine, Seoul, South Korea, Yong-In Si, Kyonggi-do, Republic of Korea; Yonsei University School of Medicine, Seoul, Seoul-t'ukpyolsi, Republic of Korea; Yonsei University College of Medicine, seoul, Seoul-t'ukpyolsi, Republic of Korea; Yonsei University College of Medicine, seoul, Seoul-t'ukpyolsi, Republic of Korea; Yonsei University College of Medicine, seoul, Seoul-t'ukpyolsi, Republic of Korea; Yonsei University College of Medicine, seoul, Seoul-t'ukpyolsi, Republic of Korea; Yonsei University College of Medicine, seoul, Seoul-t'ukpyolsi, Republic of Korea; Division of Infectious Diseases, Department of Internal Medicine, Yonsei University College of Medicine, Seoul, Seoul-t'ukpyolsi, Republic of Korea; Yonsei University College of Medicine, seoul, Seoul-t'ukpyolsi, Republic of Korea; Division of Infectious Diseases, Department of Internal Medicine, Yonsei University College of Medicine, Seoul, Seoul-t'ukpyolsi, Republic of Korea; Hanyang University College of Medicine, Yongin, Kyonggi-do, Republic of Korea; Dept. of Biomedical Systems Informatics, Yonsei University College of Medicine, Seoul, South Korea, Yong-In Si, Kyonggi-do, Republic of Korea

## Abstract

**Background:**

Atrial fibrillation (AF) is known as a poor prognostic factor in sepsis patients. Recently, research has been conducted on the clinical effects of AF burden, however, there have been no studies on the effect of AF burden in patients with sepsis. We aim to examine the effect of AF burden on in-hospital mortality in patients with sepsis.

**Methods:**

This study was conducted using patient and electrocardiogram (ECG) waveform data extracted from the Medical Information Mart for Intensive Care III (MIMIC-III) database, which was organized by the Beth Israel Deaconess Medical Center Intensive Care Unit (ICU) from 2001 to 2012. Sepsis was identified by ICD-9 code, and patients 18 years and older with lead II ECG waveform data were included in the analysis. AF classification convolutional neural network models were trained using a public ECG dataset (PTB-XL and 2017 PhysioNet/CinC Challenge), and external validation was performed using another public ECG dataset (Shaoxing and Ningbo hospital ECG database). ECG waveforms were analyzed using the SE-ResNet-34 model, which showed the best performance in external validation. Signal quality assessment was performed, and waveforms with poor signal quality were excluded. The entire waveform data of each patient was divided into 10-second segments, and the model was applied to each segment. AF burden was calculated as the ratio of AF waveforms to the total number of waveforms. The effect of AF burden on in-hospital mortality was analyzed with multivariate logistic regression, and clinical variables measured first after admission to the ICU were included in the analysis as covariates.

**Table 1.**
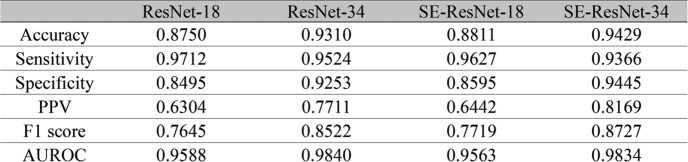
External validation results of atrial fibrillation classification models.

Model performance was evaluated with cut-off value 0.5. PPV, positive predictive value; AUROC, area under receiver operating characteristic curve.

**Results:**

A total of 795 patients with sepsis were included in the analysis. Median age was 67 (IQR, 54-79) years. Total 270 (34.0%) were in non-survivor group, and median AF burden was 2.86% (IQR 0.28%-22.07%). AF burden was significantly higher in non-survivor group (8.79% vs 1.62%, *p* ≤0.001). In the multivariate analysis, AF burden (10-unit increase) (OR, 1.13; 95% CI, 1.06-1.21; *p* ≤0.001), higher level of phosphate (OR, 1.17; 95% CI, 1.01-1.35; *p*=0.035), and higher level of lactate (OR, 1.42; 95% CI, 1.26-1.61; *p* ≤0.001) were identified as risk factors for in-hospital mortality.

**Table 2.**
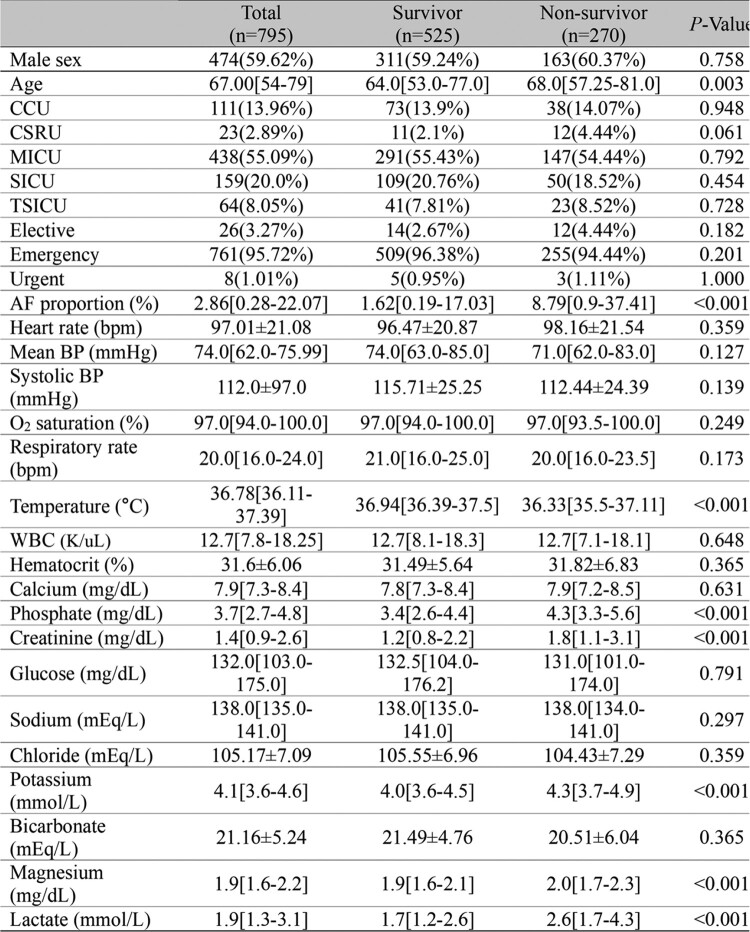
Baseline characteristics and laboratory findings of patients with sepsis. CCU, Coronary Care Unit; CSRU, Cardiac Surgery Recovery Unit; MICU, Medical Intensive Care Unit; SICU, Surgical Intensive Care Unit; TSICU, Trauma Surgical Intensive Care Unit; AF, atrial fibrillation; BP, blood pressure; WBC, white blood cell.

**Figure 1.**
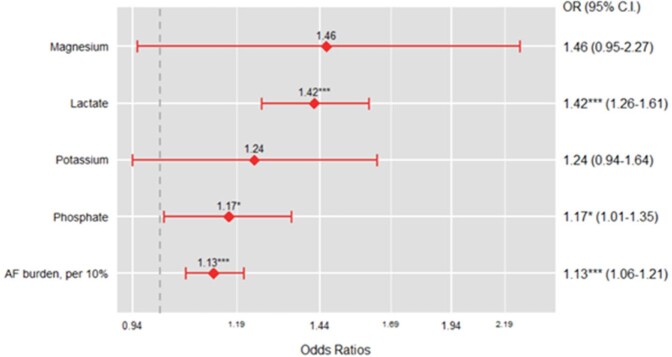
Risk factors for in-hospital mortality in patients with sepsis. * p < 0.05, ** p < 0.01, *** p < 0.001. AF, Atrial Fibrillation; OR, Odds Ratio; C.I., Confidence Interval.

**Conclusion:**

Higher AF burden is associated with in-hospital mortality in patients with sepsis.

**Disclosures:**

**All Authors**: No reported disclosures

